# Supplementary material for: Quantitative analysis of trabecular bone tissue cryosections via a fully automated neural network-based approach
Source: PLoS One. 2024 Apr 16;19(4):e0298830. doi: 10.1371/journal.pone.0298830 (PMC11020490; doi:10.1371/journal.pone.0298830)
Supplement: Attachment — Submitted filename: Response to Reviewers.docx [file pone.0298830.s001.docx]

Response to the academic Editor & Reviewers

1. Please ensure that your manuscript meets PLOS ONE's style requirements, including those for file naming. The PLOS ONE style templates can be found at 
<https://journals.plos.org/plosone/s/file?id=wjVg/PLOSOne_formatting_sample_main_body.pdf> and 
<https://journals.plos.org/plosone/s/file?id=ba62/PLOSOne_formatting_sample_title_authors_affiliations.pdf>

Thank you for the remark. We changed the manuscripts overall style to fit the requirements for PLOS ONE. We removed Institutional addresses, changing citational style and headline structure.

2. Please note that PLOS ONE has specific guidelines on code sharing for submissions in which author-generated code underpins the findings in the manuscript. In these cases, all author-generated code must be made available without restrictions upon publication of the work. Please review our guidelines at <https://journals.plos.org/plosone/s/materials-and-software-sharing#loc-sharing-code> and ensure that your code is shared in a way that follows best practice and facilitates reproducibility and reuse.

We uploaded the used code, installation instructions and all required information to reproduce created results to a github repository. <https://github.com/ChrisPohl/Bone-cryosection-segmentation>

It is a freely accessible platform, so other researchers are able to replicate the experiments without any problems. Image data can be found over github and is deposited on an accessible google drive folder.

*3. Thank you for stating the following financial disclosure: 
 "This work was funded by the ESRE, ESF and ELER as well as the Ministry of education, science, and culture of Mecklenburg Western Pomerania ‘Exzellenzforschungsprogramm’ and is part of the HOGEMA joint research project under the sponsorship contract ESF/14-BM-A55-0015/18."*
 
Please state what role the funders took in the study.  If the funders had no role, please state: "The funders had no role in study design, data collection and analysis, decision to publish, or preparation of the manuscript." 
If this statement is not correct you must amend it as needed. 
Please include this amended Role of Funder statement in your cover letter; we will change the online submission form on your behalf.

The statement: "The funders had no role in study design, data collection and analysis, decision to publish, or preparation of the manuscript." is true. Therefore, we included it in the cover letter, as you suggested.

4. Thank you for stating the following in the Acknowledgments Section of your manuscript: 
"This work was funded by the ESRE, ESF and ELER as well as the Ministry of education, science, and culture of Mecklenburg Western Pomerania ‘Exzellenzforschungsprogramm’ and is part of the HOGEMA joint research project under the sponsorship contract ESF/14-BM-A55-0015/18.
We thank Kirsten Kesselring, Antje Janetzko (Department of General Surgery, Visceral, Thoracic and Vascular Surgery, University Medical Center Greifswald) and Daniel Wolter (Department of Oral, Maxillofacial and Plastic Surgery, University Medical Center Rostock) for their excellent technical support.
We also want to express our thanks to the Institute of Anatomy, Rostock University Medical Centre for providing us with the bone samples used in this publication.
Image 1 was created with [BioRender.com](http://BioRender.com)."

We note that you have provided funding information that is not currently declared in your Funding Statement. However, funding information should not appear in the Acknowledgments section or other areas of your manuscript. We will only publish funding information present in the Funding Statement section of the online submission form. 
Please remove any funding-related text from the manuscript and let us know how you would like to update your Funding Statement. Currently, your Funding Statement reads as follows: 
"This work was funded by the ESRE, ESF and ELER as well as the Ministry of education, science, and culture of Mecklenburg Western Pomerania ‘Exzellenzforschungsprogramm’ and is part of the HOGEMA joint research project under the sponsorship contract ESF/14-BM-A55-0015/18."

Please include your amended statements within your cover letter; we will change the online submission form on your behalf.

We have not received any additional funding except the mentioned. To further differentiate funding from acknowledgement, we removed the funding information from the manuscript altogether. Further information in this part of the manuscript have nothing to do with funding and are just expressions of acknowledgement to the named people.

“We thank Kirsten Kesselring, Antje Janetzko (Department of General Surgery, Visceral, Thoracic and Vascular Surgery, University Medical Center Greifswald) and Daniel Wolter (Department of Oral, Maxillofacial and Plastic Surgery, University Medical Center Rostock) for their excellent technical support.
We also want to express our thanks to the Institute of Anatomy, Rostock University Medical Centre for providing us with the bone samples used in this publication.
Image 1 was created with [BioRender.com](http://BioRender.com)."

5. We note that you have stated that you will provide repository information for your data at acceptance. Should your manuscript be accepted for publication, we will hold it until you provide the relevant accession numbers or DOIs necessary to access your data. If you wish to make changes to your Data Availability statement, please describe these changes in your cover letter and we will update your Data Availability statement to reflect the information you provide.

Our image and created classifier data are available at google drive under the following link: <https://drive.google.com/drive/folders/1_EnP-1o8nJR5WGvvrgxPYv2ielXNLhii?usp=sharing>

A link to it can also be found on the github repository, which holds all applied code and installation instructions.

We would be happy if you could change the data availability in the online form.

6. Please amend your authorship list in your manuscript file to include author Dr. Michael Schlosser.

Thank you for this remark. In response we included Dr. Schlosser in the manuscript author list.

7. Please include captions for your Supporting Information files at the end of your manuscript, and update any in-text citations to match accordingly. Please see our Supporting Information guidelines for more information: <http://journals.plos.org/plosone/s/supporting-information>.

We chose to rearrange all additional information into a single github repository for user convenience. So, we removed the supporting information altogether from the manuscript and compiled it in one convenient place.

8. Please review your reference list to ensure that it is complete and correct. If you have cited papers that have been retracted, please include the rationale for doing so in the manuscript text, or remove these references and replace them with relevant current references. Any changes to the reference list should be mentioned in the rebuttal letter that accompanies your revised manuscript. If you need to cite a retracted article, indicate the article’s retracted status in the References list and also include a citation and full reference for the retraction notice.

We reviewed our References and found no retractions or major corrections among them. We Found two minor corrections in the following papers:

**Reference 30: Pathologists’ diagnosis of invasive melanoma and melanocytic proliferations: observer accuracy and reproducibility study**

In this article (BMJ 2017; 357, doi: 10.1136/bmj.j2813), the author affiliation for Raymond L Barnhill should have stated [^2^ Department of Pathology, Institut Curie, University of Paris Descartes Faculty of Medicine, Paris, France ] not [^2^ Department of Pathology, Institut Curie Institute Hospital, University of Paris Descartes Faculty of Medicine University, Paris, France].

**Reference 32: Dermatologist-level classification of skin cancer with deep neural networks**

In the Acknowledgements section of this Letter, the sentence: “This study was supported by the Baxter Foundation, California Institute for Regenerative Medicine (CIRM) grants TT3-05501 and RB5-07469 and US National Institutes of Health (NIH) grants AG044815, AG009521, NS089533, AR063963 and AG020961 (H.M.B.)” should have read: “This study was supported by funding from the Baxter Foundation to H.M.B.” Furthermore, the last line of the Acknowledgements section should have read: “In addition, this work was supported by a National Institutes of Health (NIH) National Center for Advancing Translational Science Clinical and Translational Science Award (UL1 TR001085). The content is solely the responsibility of the authors and does not necessarily represent the official views of the NIH.” The original Letter has been corrected online.

Both corrections are just of organizational nature and do not affect the scientific conclusions of the papers. Accordingly, we left the literature list unchanged.

In addition we included two further references:

27. van der Walt S, Schönberger JL, Nunez-Iglesias J, Boulogne F, Warner JD, Yager N, et al. scikit-image: image processing in Python. PeerJ. 2014; 2:e453. Epub 2014/06/19. doi: 10.7717/peerj.453 PMID: 25024921.

28. Paszke A, Gross S, Massa F, Lerer A, Bradbury J, Chanan G, et al. PyTorch: An Imperative Style, High-Performance Deep Learning Library. ; 03.12.2019.

Both are used in the method part to give credit to the used tools.

Response to Reviewer #1

-In line 111, include period before “Lately”
-In line 114, remove period after “analysis”
-In line 116, remove network after “neural”
-In line 155, replace “D” with “d” in Decalcification

Thank you for pointing out these errors. We addressed all remarks and corrected the manuscript accordingly. We also changed a few further points for consistency and correctness.

-In discussion section, it is mentioned that during the processing bone marrow was washed away therefore not analyzed, so can you suggest any modification in the protocol followed by you, so that bone marrow could also be imagined?

In our research questions the need for bone marrow analysis was absent, therefore it is not included in the protocol. However, we included a section suggesting protocol alterations to include marrow as a possible analysis target.

*“Unfortunately, during processing most of the bone marrow is washed out and analysis of it is therefore not achievable. To retain marrow structures, we recommend a shorter fixation in bousin solution, the creation of sections with 3-4µm thickness resulting in longer decalcification times and very gentle washing steps.”*

Response to Reviewer #2

Methodology and Protocols:

Could you provide further details on the specific steps involved in the cryosection protocol developed for trabecular bone tissue?

We addressed the request by adding the following details regarding sectioning protocol:

*“It is helpful to move the sample in the embedding medium until no further air bubbles emerge from it. Air bubbles create differences in resistance during sectioning and can therefore cause ruptures.”*

*“If the sample is of cubic shape we recommend to align the blade with the tissue so that the initial contact-area of blade to tissue is maximized.”*

Comparative Analysis:

What were the specific strengths and limitations observed in the comparison of automated image analysis methods versus manual analysis?

We addressed the request by adding, highlighting and expanding the following parts:

“Automated analysis offers great possibilities to handle large amounts of image data quantitatively in a fraction of the time manual analysis would require, while maintaining objective, investigator independent results. However automated processes are difficult to properly establish, especially for complex questions and difficult image data. It is also unable to notice spontaneous or unexpected findings, it is incapable of producing the same variability and modularity as a manual investigation would.”

*“This limitation in application might be due to the challenging code-heavy approach and the hard to access and even harder to navigate software that is necessary to utilize this approach.”*

Software and Tools:

How was the QuPath software utilized in the image analysis process, and what specific features or tools within QuPath were instrumental in achieving the study's objectives?

We added another paragraph detailing the used features:

*“With the applied QuPath network approach we exclusively utilised QuPaths build in functions. The manual annotation functions were used to segment and create the Ground-Truths that were used to train our networks. Instrumental for creation of our results were the training of a pixel classifier by artificial neural network (ANN_MLP), and the build in multifactorial cell counting function.”*

Were there any challenges or limitations encountered while implementing the neural network-based approach using UNet? How were these challenges addressed?

We clarified the limitations and challenges UNet poses by adding to the following sentence:

*“However, it requires a lot of computational power and expertise to set up, and is therefore inaccessible for a large number of labs. While UNet is a powerful tool to reliably classify even complex image data, the network trained with ‘QuPath’ offered sufficient results for our image data.”*

Results and Validation:

Can you provide more details on the statistical methods used to validate the automated image analysis results against manual analysis? Were there any statistical tests or measures of agreement used?

In order to avoid redundancies in the figure descriptions we tried to incorporate all necessary information into the subsection “Statistical Analysis”. To clarify which statistical tests were applied for each analysis, we restructured and added further explanation into said subsection. We hope this addresses the remark sufficiently.

*“Generated data were analysed using GraphPad PRISM Version 10 (GraphPad Software, Inc., San Diego, CA, USA). Normality of all data was tested using Shapiro-Wilk test. To compare the different segmentation methods, nNot normally distributed data like Jaccard indices were analysed with Kruskal-Wallis-test followed by Dunn correction for multiple testing. For comparison of the QuPath classifier with manual analysis a Wilcoxon matched pair signed rank test was used to analyse the difference between the coefficient of variances for tissue area and cells per tissue area, whereas, where data was not normally distributed. A paired t-test was used to check for significant differences between the normally distributed data, like cell counts, area in pixel, area in mm2 and counts per mm2. Not normally distributed data like Jaccard indices were analysed with Kruskal-Wallis-test followed by Dunn correction for multiple testing. For all statistical analyses, a value of p < 0.05 was considered significant (*p≤0.05; **p≤0.01; ****p≤0.0001). Further information is provided in the respective figure legends.”*

In what specific aspects did the automated analysis show variations or discrepancies compared to manual analysis, if any?

The automated showed just very slight differences in performance. We further explained and highlighted these differences by adding the following to the manuscript:

*“In most cases the automatically segmented result replicated the manual data highly, differences mostly stem from tissue edge pixel or small artifact misclassification.”*

The variance in cell count per tissue area was however observed to be significantly lower in the network-based approach compared to a manual approach as mentioned in:

*“To gain an insight into the differences between both approaches regarding accuracy, we evaluated the coefficient of variance. While the measurement of tissue area revealed no significant differences between the approaches (Figure 4C), we saw highly significant differences in numbers of cell count per tissue area (P<0.0001, Figure 4C).”*

Limitations and Future Directions:

What future applications or enhancements do you envision for this methodology, beyond trabecular bone tissue analysis, and how might these be addressed?

To explain further appliances for the presented method we added the following:

*“Although we used this analysis protocol for bone tissue specifically, it works for a wide variety of different tissues and scientific questions. Presented method can easily be used to segment a number of different tissues from their respective background, it could even be further enhanced to separate and identify multiple tissue types from another.”*

Conclusion and Implications:

Can you expand upon the implications of the study's findings for the field of histological analysis and its potential impact on research practices? How might the study's results influence the adoption of automated image analysis methods in histology, considering the current reliance on manual analysis?

To further place the importance of our protocol and quantitative histology in regard to network-based analysis we added the following sentences to our conclusion.

*“We were able to create a reliable cryosection protocol for trabecular bone tissue sections, and combined it with a functional fully automated image analysis approach.* *When compared with other automated methods as well as with a manual approach, it showed satisfying results that matched the manual gold standard, therefore creating a reliable alternative. Though manual analysis methods will remain the gold standard in quantitative histology for now, the possibility to change is given with innovative tools and easily accessible and navigable neural networking software**. To validate histology for quantitative analysis, it needs to be able to objectively quantify large datasets. In the past this was always a limitation to classical histology because of the connected workload. The need for quantifiable results from actual imaging of tissue and cells alike is still rising and deep analysis-based protocols offer solutions to connected problems. Hence, we are positive that network-based analysis will gain more importance with time, based on its flexibility in use, its ability to handle large amounts of data in minimal time, as well as its reliability and will therefore continue to find its way into quantitative histology and even more scientific fields.”*
